# RN-Autoencoder: Reduced Noise Autoencoder for classifying imbalanced cancer genomic data

**DOI:** 10.1186/s13036-022-00319-3

**Published:** 2023-01-30

**Authors:** Ahmed Arafa, Nawal El-Fishawy, Mohammed Badawy, Marwa Radad

**Affiliations:** Faculty of Electronic Engineering, Menoufia University, El-Gish Street, Box No. 32951, Menouf, Menoufia Egypt

**Keywords:** RN-Autoencoder, Cancer Classification, Gene Expressions, Imbalanced Classification, RN-SMOTE, Dimensionality Reduction

## Abstract

**Background:**

In the current genomic era, gene expression datasets have become one of the main tools utilized in cancer classification. Both curse of dimensionality and class imbalance problems are inherent characteristics of these datasets. These characteristics have a negative impact on the performance of most classifiers when used to classify cancer using genomic datasets.

**Results:**

This paper introduces Reduced Noise-Autoencoder (RN-Autoencoder) for pre-processing imbalanced genomic datasets for precise cancer classification. Firstly, RN-Autoencoder solves the curse of dimensionality problem by utilizing the autoencoder for feature reduction and hence generating new extracted data with lower dimensionality. In the next stage, RN-Autoencoder introduces the extracted data to the well-known Reduced Noise-Synthesis Minority Over Sampling Technique (RN- SMOTE) that efficiently solve the problem of class imbalance in the extracted data. RN-Autoencoder has been evaluated using different classifiers and various imbalanced datasets with different imbalance ratios. The results proved that the performance of the classifiers has been improved with RN-Autoencoder and outperformed the performance with original data and extracted data with percentages based on the classifier, dataset and evaluation metric. Also, the performance of RN-Autoencoder has been compared to the performance of the current state of the art and resulted in an increase up to 18.017, 19.183, 18.58 and 8.87% in terms of test accuracy using colon, leukemia, Diffuse Large B-Cell Lymphoma (DLBCL) and Wisconsin Diagnostic Breast Cancer (WDBC) datasets respectively.

**Conclusion:**

RN-Autoencoder is a model for cancer classification using imbalanced gene expression datasets. It utilizes the autoencoder to reduce the high dimensionality of the gene expression datasets and then handles the class imbalance using RN-SMOTE. RN-Autoencoder has been evaluated using many different classifiers and many different imbalanced datasets. The performance of many classifiers has improved and some have succeeded in classifying cancer with 100% performance in terms of all used metrics. In addition, RN-Autoencoder outperformed many recent works using the same datasets.

## Introduction

Cancer is a serious disease and is currently one of the main causes of death worldwide. The classification of cancer using gene expression datasets is one of the best and most accurate methods as a result of the knowledge acquired through these datasets [1]. This is because gene expression tracks the expression levels of thousands of genes simultaneously [2]. However, because they contain thousands of genes and a small number of samples, they are high-dimensional datasets. So, these datasets are computationally unmanageable as a result of their dimensionality [3]. Within these datasets, there are four categories of features: irrelevant, relevant with redundant, relevant without redundant and strongly relevant features. The best feature set includes only the last two categories [4]. On the contrary, irrelevant and redundant features always increase the training time significantly and negatively affect the performance of the used classifier. So, these features should be removed from the dataset before training the classifier using the dataset [5]. Also, the existence of noisy features as an inherent property of the data reduces the performance of the underlying classifier [6]. Due to the dimensionality and its subsequent problems, a preliminary pre-processing step is required before introducing the high-dimensional dataset to the classifier. This can be achieved by utilizing two common approaches: feature extraction and feature selection techniques.

Feature selection selects the subset representing the most informative and relevant features from the original high-dimensional data [7, 8]. Many feature selection methods have been introduced to remove the irrelevant and redundant features to reduce the computation requirements, tackle the curse of dimensionality and provide a more efficient understanding of the high-dimensional data. This will improve the performance of various classifiers on high-dimensional datasets [9]. These methods have many taxonomies according to different criteria. They can be classified into supervised, unsupervised and semi-supervised according to the utilization of the class label. Also, they can be classified into filter, wrapper and embedded models according to their relationship with learning methods. Also, according to the evaluation criteria, they can be classified into correlation, euclidean distance, consistency, dependence and information measure. Another classification is to classify them according to the search strategies into forward increase, backward deletion, random and hybrid models. Finally, they can be classified into feature rank (Weighting) and subset selection models according to the type of output [10–12]. Also, the feature selection process can be considered as an optimization problem and hence many optimization techniques such as Particle Swarm Optimization (PSO) and Genetic Algorithms (GA) can be utilized for feature selection [13, 14].

Feature Extraction creates a new set of features that replace the original high-dimensional dataset. It is needed for many different reasons including data compression, noise removal, data visualization, increasing the training speed, analysis of parameters and tackling the curse of dimensionality [15]. While feature extraction compresses new features more efficiently, the new extracted features may lose their meaning [16, 17]. Feature extraction methods are classified as linear and non-linear methods. Some linear methods are based on variance total contribution ratios such as Principal Component Analysis (PCA), Linear Discriminant Analysis (LDA) and Factor Analysis (FA). Other linear methods are based on Independent Component Analysis (ICA) and others are based on Singular Value Decomposition (SVD) and Multidimensional Scaling Analysis (MDS). Non-linear methods include kernel-optimized functions such as Kernel-based Principal Component Analysis (KPCA), Nonnegative Matrix Factorization (NMF), Wavelet Transformation (WT), Locally Linear Embedding (LLE) and deep learning approaches including deep autoencoders [18].

Imbalanced class distribution in cancer genomic datasets is another challenging problem that has a negative impact on the performance of most classifiers. SMOTE is a well-known oversampling technique that is widely used to solve the class imbalance problem by synthesizing new samples for minor class [19]. SMOTE tackles the overfitting problem caused by other oversampling techniques such as random oversampling because it does not repeat existing samples. However, SMOTE is sensitive to the high dimensionality problem [20]. Also, it neglects the major class neighbours resulting in more overlapping between major and minor classes [21]. Moreover, SMOTE generates noisy samples which negatively affect the performance of the used classifier [22, 23]. Many SMOTE extensions have been proposed to overcome these limitations. RN-SMOTE is one of the recent extensions that utilize the Density-Based Spatial Clustering of Applications with Noise (DBSCAN) algorithm to detect and remove noise after oversampling the imbalanced dataset through SMOTE [24]. Another extension is the Limiting Radius SMOTE (LR-SMOTE) which utilizes the Support Vector Machines (SVM) and k-means to remove the noise in the original data set then oversamples the data using SMOTE to generate new samples which are filtered based on the nearest neighbours [25].

This paper introduces a new model for cancer classification using high dimensional imbalanced gene expression datasets that combines autoencoders for feature extraction and RN-SMOTE for handling class imbalance problem.

The main contributions of this paper include the following:

- Introduce RN-Autoencoder that utilizes the autoencoder for feature reduction and RN-SMOTE for handling class imbalance to handle the challenges of gene expressions.
- Evaluate RN-Autoencoder using several classifiers and different imbalanced datasets using various metrics.
- Compare the performance of the classifiers under RN-Autoencoder with their performance under both original and extracted data.
- Compare the performance of RN-Autoencoder with the performance of the current state of art.

The rest of this paper is organized as follows: Related work section reviews the related work. Material and methods section explains the proposed model by firstly introducing both the RN-SMOTE and autoencoders. Then, RN-Autoencoder is described in detail.  Results and discussion section shows the experiments and discusses the obtained results. Finally, the last section is the conclusion and future work.

## Related work

Many studies have been done to classify cancer using different types of datasets with different characteristics and dimensionalities. Gene expressions are considered one of these datasets, which are characterized by their high dimensionality. While high dimensionality presents a serious challenge to existing machine learning methods, some recent studies deal with gene expression datasets without feature reduction using different strategies. Li et al. [26] introduced a model for classifying gene expression datasets by first balancing them with SMOTE oversampling technique. The resampled data is then introduced to a Stochastic Gradient Descent (SGD) based L2-SVM classifier. This model was evaluated using four different datasets. The resulting accuracy was estimated at 93.1, 93.10, 83.7 and 85.4% for Leukemia, Myelodysplastic Syndrome (MDS), Single Nucleotide Polymorphism (SNP) and colon from Gene Expression Omnibus (GEO) repository respectively. Kakati et al. [27] introduced DEGnext that based on transfer learning and CNN to improve the classification performance of gene expression datasets. DEGnext can classify differentially expressed genes (DEGs) as upregulating genes and downregulating genes in cancer genome datasets. DEGnext has been trained and evaluated using 17 different datasets from The Cancer Genomic Atlas (TCGA) repository, resulting in Receiver Operating Characteristic (ROC) scores between 88 and 99%. Dai et al. [28] built their cancer classification model based on the correlation between gene expression samples. This model is based on a residual graph convolutional network and a sample similarity network. In the first stage, a sample similarity matrix is computed based on the Pearson correlation coefficient of the gene expression data, yielding an adjacency matrix. In the second stage, the adjacency matrix and the input features are introduced to a residual graph convolutional network for classification. This model was applied to breast invasive carcinoma (BRCA), Glioblastoma Multiforme (GBM) and lung cancer (LUNG). It achieved an accuracy of 82.58, 85.13 and 79.18% for the BRCA, GBM and LUNG datasets respectively.

Other studies employ a single feature selection method to enhance the performance of their machine learning models on gene expression datasets. Mohammed et al. [29] introduced a one-dimensional convolutional neural network (1D-CNN) model for cancer classification in which LASSO regression has been utilized for feature selection. This model has been trained and evaluated using five RNASeq datasets among the TCGA pan-cancer datasets. This model scored an average performance estimated at 99.55, 99.29 and 99.42% in terms of precision, recall and F1-Score respectively. Menaga et al. [30] used wrapper method for feature selection in their introduced model for classifying cancer from colon and leukemia datasets. the selected features have been classified using Deep Recurrent Neural Network (Deep RNN), which is optimized by the utilization of Fractional-Atom Search Algorithm (FASO). This model achieved an accuracy estimated at 92.87 and 92.82% for the colon and leukemia datasets respectively. Al Mamun et al. [31] proposed the use of Concrete Autoencoder (CAE) [32] to discover the critical Long non-coding RNA (lncRNAs) that can identify the origin of 12 different types of cancers from TCGA repository. They proposed multi-run CAE (mrCAE) to identify a stable set of top-ranking 128 lncRNAs. Their model mrCAE could identify the origin of 12 different cancers with an accuracy of 95%.

Combining multiple feature selection methods is the main strategy of other researchers for efficiently dealing with gene expression datasets. Majumder et al. [33] combined Analysis of Variance (ANOVA) and Information Gain (IG) for feature selection from four different gene expression datasets. Then, Multilayer Perceptron (MLP), 1DCNN and 2DCNN deep learning architectures have been used for classifying the selected features. Based on the used dataset and the feature selection, the MLP model resulted in an accuracy that ranges from 77 to 95%, the 1DCNN resulted in an accuracy that ranges from 77 to 100% and the 2DCNN resulted in an accuracy that ranges from 77 to 90%. Saberi-Movahed et al. [34] propounded a novel feature selection approach called Dual Regularized Unsupervised Feature Selection Based on Matrix Factorization and Minimum Redundancy (DR-FS-MFMR). Their approach uses matrix factorization and subspace learning strategies to drive useful features. DR-FS-MFMR has been evaluated on nine different gene expression datasets. Bustamam et al. [35] proposed two stages of feature selection for their lung cancer diagnostic tool. They use support vector machine-recursive feature elimination SVM-RFE to prefilter the genes. The selected genes are chosen again using Artificial Bee Colony algorithm ABC, an optimization method proposed based on the social behavior of honeybees as they search for high-quality food sources. They built their tool using SVM. The accuracy of SVM with RFE-ABC as the feature selection method reached 98% with 100 best features for the Michigan lung cancer dataset [36] and 97% with 70 best features for the Ontario lung cancer dataset [37].

On the other point of view, feature extraction methods can support cancer classification models on gene expression datasets. Devendran et al. [38] reduced the high dimensionality of the genomic data by utilizing Probabilistic Principal Component Analysis (PPCA) to enhance the performance of their cancer classification proposal. Following that, the resulting features were classified using a CNN, with the weights optimized using Fractional Biogeography-Based Optimization (FBBO). This proposal is evaluated using both the colon and leukemia datasets, resulting in an accuracy estimated at 98% for the two datasets. Majji et al. [39] used non-negative matrix factorization for feature extraction in their cancer classification model. The resulting features are introduced to a recurrent neural network based on JayaAnt lion optimization for classification. This model is trained and evaluated using four datasets, which are AP_Colon_Kidney [40], AP_Breast_Ovary [41], AP_Breast_Colon [42] and AP_Breast_Kidney [43] datasets. This model resulted in a maximum accuracy, sensitivity and specificity of 95.97, 95.95 and 96.96%.

Combining feature selection and feature extraction methods provides a promising solution for the high dimensionality curse of gene expression datasets. Pandit et al. [44] introduced a hybrid approach for cancer classification using genomic datasets. In this approach, the gene expressions data were first preprocessed using an adaptive filter and then clustered using an improved binomial clustering approach. In the next step, new data is extracted using the multifractal Brownian motion method. Then, the most relevant features are selected using an improved cuckoo search optimization. After that, the selected features are introduced to a wavelet-based Convolutional Neural Network (CNN) for classification, resulting in an accuracy estimated at 98.36, 98.12, 98.55, 97.70 and 95.30% for ovarian, breast, colon, leukemia and prostate cancer datasets respectively. Uzma et al. [45] introduced the Gene-encoder to classify cancer based on multiple feature reduction techniques represented in two stages. In the first stage, it uses an ensemble of three feature reduction techniques, including Principal Component Analysis (PCA), correlation and spectral feature selection. In the second stage, the Genetic Algorithm (GA) is combined with autoencoder and K-means clustering to select a set of features and then evaluate them. The resulting subset of features has been classified using SVM, Kth Nearest Neighbours (KNN) and Random Forest (RF) classifiers. Gene-encoder has been evaluated using six different gene expressions datasets, including the colon, leukemia and DLBCL datasets. The performance of SVM outperformed KNN and RF for colon, leukemia and DLBCL datasets and scored an accuracy estimated at 97, 84 and 90% respectively.

Besides high-dimensional gene expressions datasets, we also included recent studies for cancer classification using small-dimensional datasets, namely Wisconsin Diagnostic Breast Cancer (WDBC) dataset. It contains 30 features computed from a digitized image of a fine needle aspirate (FNA) of a breast mass [46]. We use it to evaluate our proposal in light of the recent researches.

Just like gene expression datasets, different strategies of feature reduction have been utilized to enhance breast cancer classification accuracy based on WDBC dataset. Multiple feature selection methods have been used to build Meta Health Stack by Samieinasab et al. [47]. In this model, the Extra Trees classifier is used to combine features resulting from the Variance Inflation Factor, Pearson’s Correlation and Information Gain. The resulting features are introduced to a stacked ensemble classifier composed of bagging, boosting and voting classifiers. This model resulted in a performance estimated at 97% in terms of F1-score and 98% in terms of precision. On the other hand, Singh et al. [48] used PCA as a feature extraction method in their study to evaluate the performance of many different classifiers for cancer classification using the WDBC and the Surveillance, Epidemiology and End Results (SEER) datasets [49]. This evaluation resulted in maximal performance using WDBC in terms of the accuracy estimated at 97.66%, obtained by SVM and RF classifiers. Also, with the SEER dataset, the maximum performance in terms of accuracy has been estimated at 99.5%, obtained by decision tree and Naïve Bayes classifiers. On the same methodology, Bacha et al. [50] used kernel principal component analysis (KPCA) for feature extraction in their breast cancer diagnosis model. They run their model on WDBC and Mammographic Image Analysis Society (MIAS) datasets. They used differential evaluation to optimize the hyperparameters of the Radial-Based Function Kernel Extreme Learning Machines (RBF-KELM), which are used as a classifier. This model resulted in a maximal performance in terms of accuracy, estimated at 91.13% with the WDBC dataset and 100% with the MIAS dataset. Table 1 lists the summary of all studies included in the related work.

**Table 1:** Summary of related work studies

Authors	Year	#Datasets	Feature Reduction	Classifier
**No Feature Reduction**				
Li et al. [26]	2022	4	-	SMOTE Resampling + L2-SVM
Kakati et al [27]	2022	17	-	Transfer learning + CNN
Dai et al. [28]	2021	3	-	ERGCN
**Single Stage Feature Selection**				
Mohammed et al. [29]	2021	5	Lasso	Staking Ensemble of CNN
Menaga et al. [30]	2021	2	Wrapper	Fractional-ASO Deep RNN
Al Mamun et al. [31]	2021	12	mrCAE	-
**Multiple Stages Feature Selection**				
Majumder et al. [33]	2022	4	ANOVA, IG	MLP, 1DCNN, 2DCNN
Saberi-Movahed et al. [34]	2022	9	DR-FS-MFMR = Matrix Factorization + Minimum Redundancy	Unsupervised clustering
Bustamam et al. [35]	2021	2	SVM-RFE + ABC	SVM
Samieinasab et al. [47]	2022	1	Ensemble (Variance Inflation Factor, Pearson’s Correlation, Information Gain)	Ensemble (Boosting, Bagging, Voting)
**Single Stage Feature Extraction**				
Devendran et al. [38]	2021	2	PPCA	FBBO + CNN
Majji et al. [39]	2021	4	Non-negative matrix factorization	JayaALO-based Deep RNN
Singh et al. [48]	2022	2	PCA	C5.0, AdaBoost, CART, GBM, NB, RF, SVM, AdaBoost
Bacha et al. [50]	2022	2	KPCA	DE-RBF-KELM
**Feature Selection + Feature Extraction**				
Pandit et.al [44]	2022	5	Binomial Clustering + Multifractional Brownian Motion + Cuckoo search optimization	Wavelet + CNN
Uzma et al. [45]	2022	5	Two stages: 1-Ensemble (PCA, Correlation, SFS) 2-Autoencoder + GA + K-means	SVM, KNN, RF

Many of the studies mentioned in related works have drawbacks regarding their performance for cancer diagnosis. This is because misclassifying a sample as "benign" while it is actually "malignant" leads to ignoring the necessary treatment of the patient, hence the exacerbation of the disease and possibly death. The performance drawbacks in these studies resulted from ignoring many important characteristics of the used datasets. Most of these studies handled dimensionality using different feature selection and extraction methods. However, many of these studies ignored the class imbalance and the existence of noise in many datasets such as the colon, leukemia, DLBCL and WDBC. These problems always have a negative impact on the classifier's performance, especially when classifying the minor class samples. So, the proposed RN-Autoencoder provides an efficient model for precise cancer diagnosis using imbalanced gene expressions datasets by utilizing autoencoder for feature reduction and RN-SMOTE for handling the class imbalance and the noise. Thereby, RN-Autoencoder successfully improves the performance of different classifiers when used with these challenged datasets.

## Material and methods

The proposed RN-Autoencoder is constructed from two stages. The first stage is the feature extraction stage, where the curse of dimensionality problem of the dataset is solved by using autoencoders. In the second stage, RN-SMOTE is utilized to handle the class imbalance in the previously extracted data. In this section, we give a brief introduction to both autoencoders and the RN-SMOTE. Then, the RN-Autoencoder is described in detail.

### Autoencoders

**Fig. 1:**
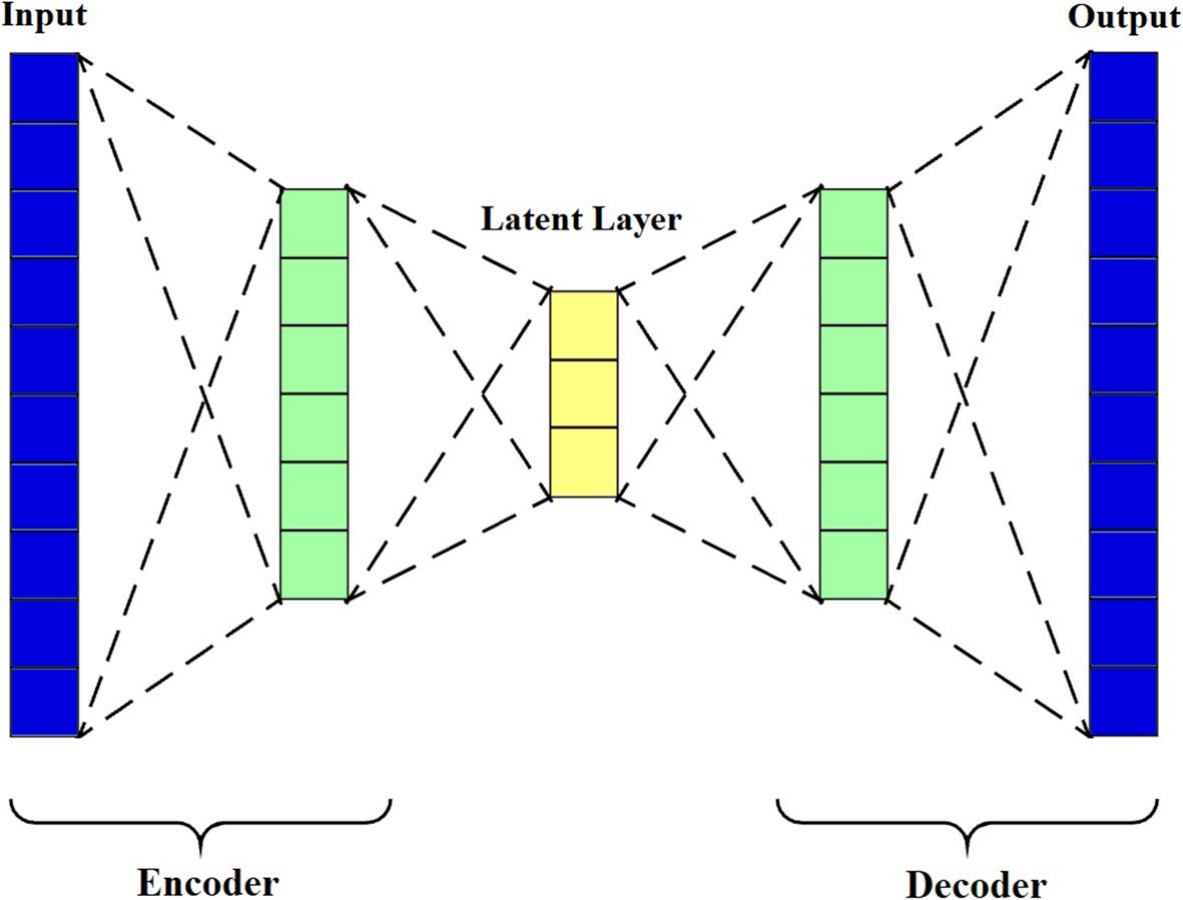
Construction of Autoencoders

The autoencoder is an unsupervised neural network with a bottleneck layer (also known as the latent layer) that represents a compressed version of the original input data [51]. Autoencoders are mainly constructed from two main parts. The first part is the encoder, which is a nonlinear transformation of the original input data to lower-dimensional data. The second part is the decoder, which reconstructs the previously encoded data to its original form [52, 53]. Figure 1 shows the basic construction of autoencoders. Autoencoders were mainly developed and used for feature extraction to reduce the high dimensionality of datasets to be ready for classification by different ML algorithms [54–57]. They have also been utilized in a variety of applications, including anomaly detection in different types of applications [58–61] and classification problems in many applications [62–64]. Since they are considered non-linear feature reduction methods, autoencoders have superior performance when compared to other linear feature reduction approaches such as PCA [65, 66].

### RN-SMOTE

RN-SMOTE is an approach for handling the class imbalance problem [24]. It first oversamples the imbalanced dataset using SMOTE by generating a set of synthetic samples from the minor class according to the equation below.

1 $$\mathbf{X}\mathbf{s}\mathbf{y}\mathbf{n}=\mathbf{X}\mathbf{i}+\mathbf{r}\mathbf{a}\mathbf{n}\mathbf{d}\left(0,1\right)\times \left|{\varvec{X}}\mathbf{i}-{\varvec{X}}\mathbf{n}\mathbf{e}\mathbf{i}\mathbf{g}\mathbf{h}\mathbf{b}\mathbf{o}\mathbf{u}\mathbf{r}\right|$$

Where ${\varvec{X}}\mathbf{i}$ is a sample from the minor class and ${\varvec{X}}\mathbf{n}\mathbf{e}\mathbf{i}\mathbf{g}\mathbf{h}\mathbf{b}\mathbf{o}\mathbf{u}\mathbf{r}$ is a randomly selected sample from the K nearest neighbours to the sample ${\varvec{X}}\mathbf{i}$ and $\mathbf{r}\mathbf{a}\mathbf{n}\mathbf{d}\left(0,1\right)$ is a random number between 0 and 1. Also, DBSCAN is a clustering algorithm that has the ability to recognize the isolated samples that are not reachable from any other point and isolates them in a separate cluster and mark it as noise. So, after oversampling the imbalanced data, RN-SMOTE detects then removes the noise from the minor class by utilizing the DBSCAN This noise may have existed in the dataset originally or as a result of the oversampling process depicted in Eq. 1.

**Fig. 2:**
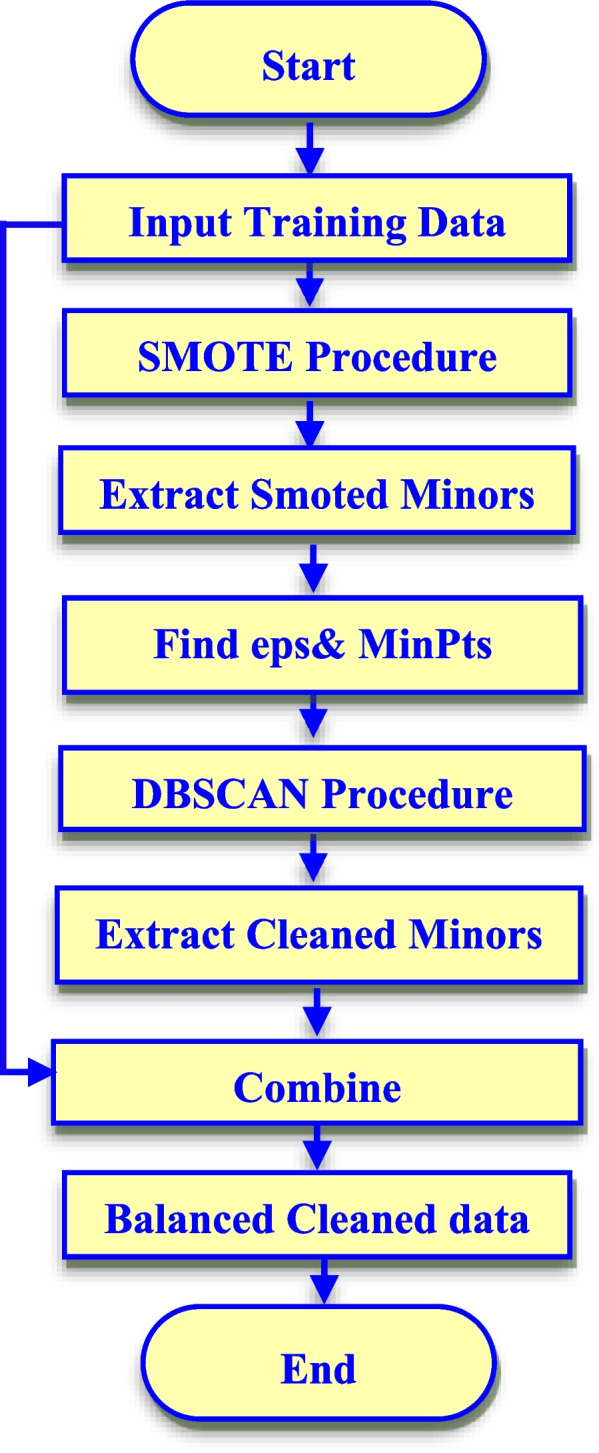
Flowchart of RN-SMOTE

Figure 2 displays the steps of the RN-SMOTE. It begins with oversampling the minor class samples in the training dataset using the SMOTE procedure. After that, the minor samples are isolated to be introduced to the DBSCAN to clean them from the noisy samples. For efficient noise detection, DBSCAN hyperparameters (eps and MinPts) are optimized by using the sorted k-distance graph. The MinPts parameter that represents the minimum number of points to create a cluster is assumed to be the natural log of the minor class samples. The eps value is decided by finding the maximum curvature point in the sorted k-distance graph. The RN-SMOTE then removes the detected noise and then combines the resultant cleaned minor samples with the original training set that includes both the original minors and major classes.

### RN- Autoencoder

**Fig. 3:**
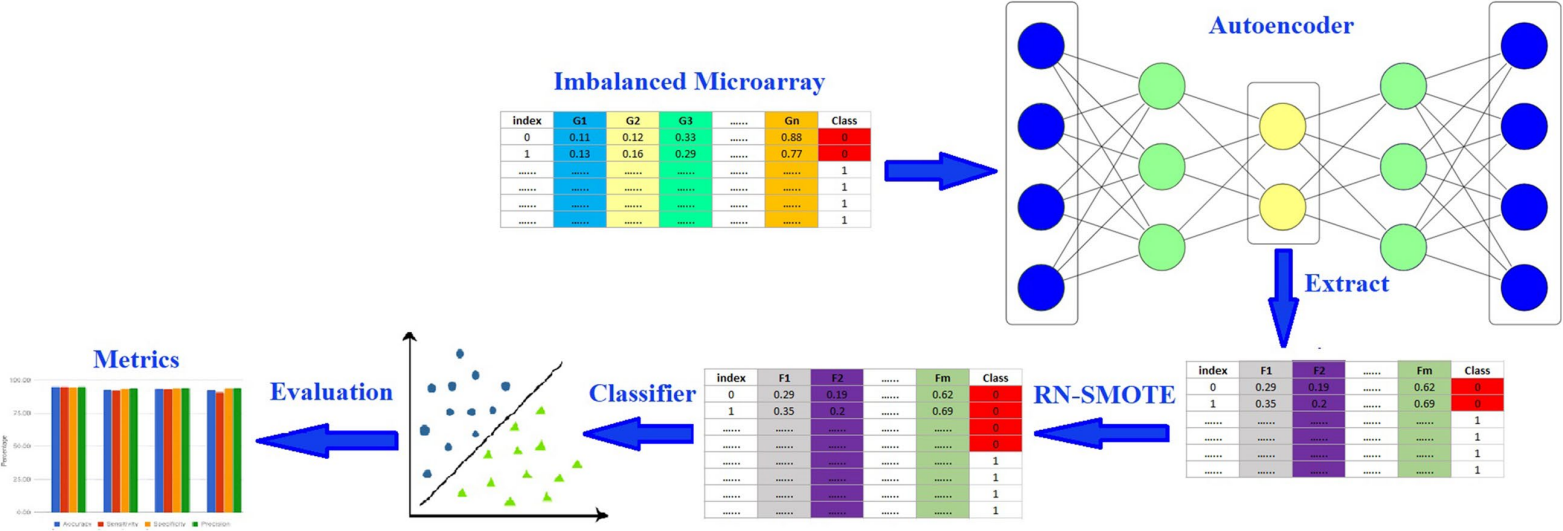
An overview of the proposed RN-Autoencoder

In this section, we describe the proposed RN-Autoencoder. The RN-Autoencoder consists of four main stages, which are the pre-processing, autoencoding, RN-SMOTE and classification stages. Figure 3 shows the sequence of these steps.

The first stage in RN-Autoencoder is the pre-processing stage, which includes stratified splitting of each input dataset into training and testing sets and then normalization techniques are applied. The Min–Max scaler is used as a normalization technique for all used datasets. It transforms the value of any feature to be in the range [0,1] according to the formula:

2 $${\varvec{x}}`=\boldsymbol{ }\frac{{\varvec{x}}-{\varvec{m}}{\varvec{i}}{\varvec{n}}\left({\varvec{x}}\right)}{{\varvec{m}}{\varvec{a}}{\varvec{x}}\left({\varvec{x}}\right)-{\varvec{m}}{\varvec{i}}{\varvec{n}}\left({\varvec{x}}\right)}$$

where ${\varvec{x}}$ is the value of the feature and ${\varvec{x}}$` is the value of the feature after the transformation.

**Fig. 4:**
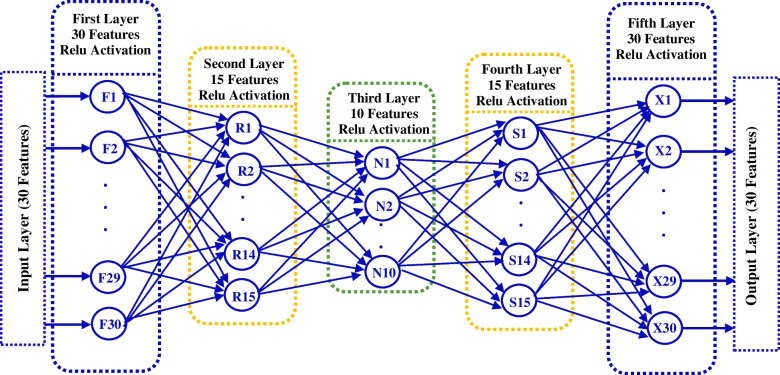
Internal Details of the WDBC dataset autoencoder

Feature reduction is a mandatory step when dealing with cancer genomic datasets to tackle the curse of dimensionality. So, in the second stage of RN-autoencoder, the autoencoder is used to reduce the dimensionality of these datasets. An autoencoder is constructed for each of the used datasets. The training set is used to build, compile and train the autoencoder, while the test set is used for its evaluation. The training process has continued until an appropriate compressed version of the training data has been extracted. At this point, the autoencoder architecture is saved to be used later to transform the test set to have the same number of features as the training set. The number of layers and nodes within each layer in each autoencoder is dependent on the used dataset, as they are different in the number of features. Figure 4 shows the number of hidden layers and the number of nodes within each layer of the autoencoder used with the WDBC dataset. It is clear that, the original 30 features in WBCD dataset can be represented will by only 10 extracted new features in the bottleneck layer. The Rectified Linear Unit (ReLU) activation function has been used in all hidden layers with all constructed autoencoders. With all datasets, the autoencoders use an exponential decay learning rate with an initial learning rate, decay steps and a decay rate value listed in Table 2. Furthermore, three datasets (colon, leukemia and DLBCL) use 100 epochs, while the WDBC and lung cancer datasets use only 50 epochs. The output of the autoencoding stage is a new extracted dataset that represents an effective compressed version of the original data.

**Table 2:** Exponential decay learning rate parameters for all datasets

Dataset	Initial Learning Rate	Decay steps	Decay Rate
Colon	**1** ${{\varvec{e}}}^{-4}$	**10,000**	**0.9**
Leukemia	**1** ${{\varvec{e}}}^{-4}$	**10,000**	**0.9**
DLBCL	**1.1** ${{\varvec{e}}}^{-4}$	**10,000**	**0.75**
Lung (Michigan)	**1** ${{\varvec{e}}}^{-4}$	**10,000**	**0.9**
WDBC	**1.1** ${{\varvec{e}}}^{-4}$	**10,000**	**0.95**

Since the compressed version generated by the autoencoder is imbalanced because the original version was imbalanced, in the third stage, RN-SMOTE is used to handle the class imbalance in the version extracted by the autoencoder. RN-SMOTE will oversample the extracted data using SMOTE and remove the resultant or originally existing noise in the minor class of the compressed version of the data resulting in a clean version of the compressed training set. This is accomplished by utilizing DBSCAN. To optimize the DBSCAN, the minimum number of points to create a class in the utilized DBSCAN is determined by the natural log (Ln) of the number of samples in the minor class which is computed using the formula:

3 $${\varvec{M}}{\varvec{i}}{\varvec{n}}{\varvec{P}}{\varvec{t}}{\varvec{s}}={\varvec{L}}{\varvec{n}}({\varvec{N}})$$

**Fig. 5:**
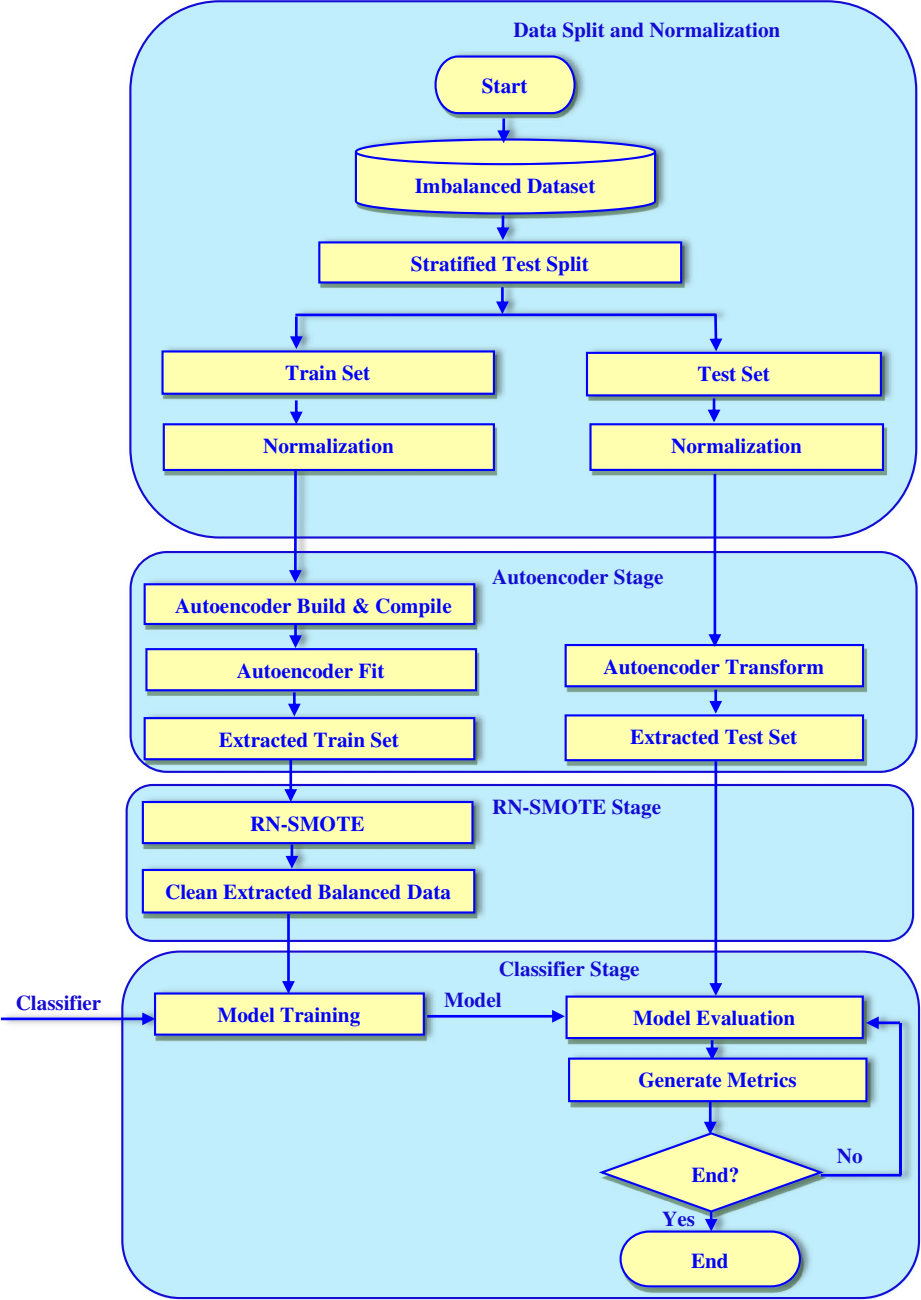
Flowchart of detailed steps of RN-Autoencoder

Finally, in the fourth stage, the clean-extracted-balanced training data resulting from the RN-SMOTE stage is introduced to many classifiers for building many models for cancer classification. The impact of the extracted data by the RN-Autoencoder on the performance of these classifiers is evaluated by using many different metrics. Figure 5 shows the detailed steps of the RN-Autoencoder.

#### Datasets and metrics

This section introduces the characteristics of the datasets and classification metrics used in this study. In the experiments that have been carried out to evaluate the proposed RN-Autoencoder model, four different imbalanced and high dimensional datasets have been used. These datasets represent the binary classification for three types of cancer, which are colon [67], leukemia [68], DLBCL [69] and Lung (Michigan). This is beside the WDBC which has also been used to evaluate the proposed model and measure its performance on small-dimension datasets. Table 3 lists the characteristics of these datasets, including the imbalance ratio, which is the ratio of the number of minor class samples to the number of major class samples.

**Table 3:** Datasets description

Dataset	#Features		#Sample	#Minors	#Majors	Imbalance Ratio (#Minors /#Majors)
Colon	**2000**	**62**		**22**	**40**	**55%**
Leukemia	**7129**	**72**		**25**	**47**	**53.19%**
DLBCL	**7070**	**77**		**19**	**58**	**32.75%**
Lung (Michigan)	**7129**	**96**		**10**	**86**	**11.62%**
WDBC	**30**	**569**		**212**	**357**	**59.38%**

True Positive (TP), False Positive (FP), True Negative (TN) and False Negative (FN) are the basic confusion matrix parameters used to compute other performance metrics such as accuracy, precision, recall and F1 scores in ML classification [70]. Also, classification tasks with imbalanced data can be evaluated using other metrics, including Matthew’s Correlation Coefficient (MCC), Cohesion’s Kappa and the Geometric-Mean (GM) score [71]. For the evaluation of the classifiers in this paper, all metrics listed in Table 4 have been used.

**Table 4:** Classification metrics

Metric	Expression	Metric	Expression
Precision	$\frac{\mathbf{T}\mathbf{P}}{\mathbf{T}\mathbf{P}+\mathbf{F}\mathbf{P}}$	**GM**	$\sqrt{\frac{{\varvec{T}}{\varvec{P}}}{{\varvec{T}}{\varvec{P}}+{\varvec{F}}{\varvec{N}}}\boldsymbol{ }\times \boldsymbol{ }\frac{{\varvec{T}}{\varvec{N}}}{{\varvec{T}}{\varvec{N}}+{\varvec{F}}{\varvec{P}}}}$
Recall	$\frac{\mathbf{T}\mathbf{P}}{\mathbf{T}\mathbf{P}+\mathbf{F}\mathbf{N}}$	**MCC**	$\frac{\mathbf{T}\mathbf{P}.\mathbf{T}\mathbf{N}-\mathbf{F}\mathbf{P}.\mathbf{F}\mathbf{N}}{\sqrt{(\mathbf{T}\mathbf{P}+\mathbf{F}\mathbf{P})(\mathbf{T}\mathbf{P}+\mathbf{F}\mathbf{N})(\mathbf{T}\mathbf{N}+\mathbf{F}\mathbf{P})(\mathbf{T}\mathbf{N}+\mathbf{F}\mathbf{N})}}$
F1	$\frac{2\mathbf{*}\mathbf{p}\mathbf{r}\mathbf{e}\mathbf{c}\mathbf{i}\mathbf{s}\mathbf{i}\mathbf{o}\mathbf{n}\mathbf{*}\mathbf{R}\mathbf{e}\mathbf{c}\mathbf{a}\mathbf{l}\mathbf{l}}{\mathbf{P}\mathbf{r}\mathbf{e}\mathbf{c}\mathbf{i}\mathbf{s}\mathbf{i}\mathbf{o}\mathbf{n}+\mathbf{R}\mathbf{e}\mathbf{c}\mathbf{a}\mathbf{l}\mathbf{l}}$	**Kappa**	$\frac{\mathbf{P}\mathbf{o}-\mathbf{P}\mathbf{e}}{1-\mathbf{P}\mathbf{e}}$
Accuracy	$\frac{{\varvec{T}}{\varvec{P}}+{\varvec{T}}{\varvec{N}}}{{\varvec{T}}{\varvec{P}}+{\varvec{T}}{\varvec{N}}+{\varvec{F}}{\varvec{P}}+{\varvec{F}}{\varvec{N}}}$		

## Results and Discussion

### Evaluation of RN-Autoencoder

The performance of different classifiers is significantly affected by dimensionality reduction, resampling and other data pre-processing techniques. So, to evaluate the effectiveness and performance of RN-Autoencoder, many classifiers have been utilized for this purpose. These classifiers are Classification and Regression Tree (CART), RF, Gradient Boost (GB), Adaptive Boosting (AdaBoost), Extreme Gradient Boosting (XGBOOST), Gaussian Naïve Bayes (GNB), Kth Nearest Neighbours (KNN), Logistic Regression with Stochastic Gradient Descent (SGD-LR), Support Vector Machines with Radial Basis Function kernel (SVM-RBF), SVM with Linear kernel (SVM-Linear) and Linear Discriminant Analysis (LDA). These classifiers have been selected because they performed well on a variety of datasets. Each classifier has been used with its default settings in python libraries. Also, all evaluation experiments have been carried out on a machine with Windows 10 operating system, 8 GB RAM and Intel I5 processor.

**Fig. 6:**
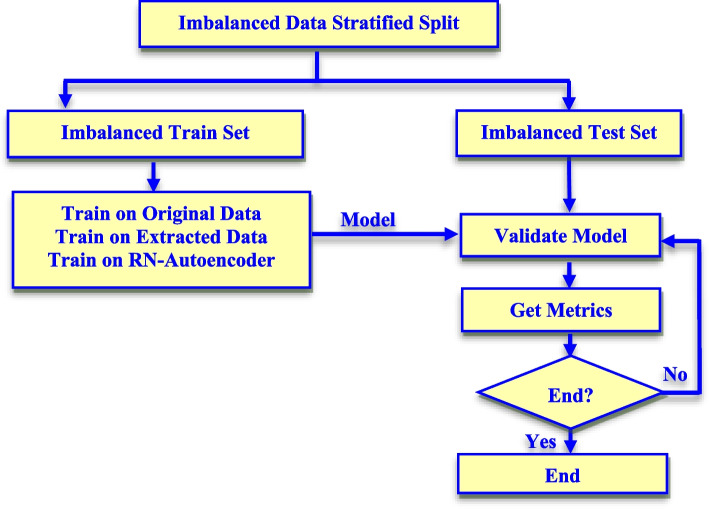
Flowchart of the evaluation of all the used classifiers

RN-Autoencoder has been evaluated by comparing the performance of each classifier in four different scenarios. The first scenario is training the classifier using the original data without any feature reduction. The second scenario is training the classifier using the original data after pre-processing it using RN-SMOTE only. The third scenario is training the classifier using the extracted data after applying the autoencoder only. Finally, the fourth scenario is training the classifier with the data obtained the after pre-processing using RN-Autoencoder. All classifiers are trained in each case on the same training set and evaluated on the same test set for a fair evaluation of the classifiers, which is shown in Fig. 6. The performance of each classifier has been measured in terms of all metrics listed in Table 4.

**Fig. 7:**
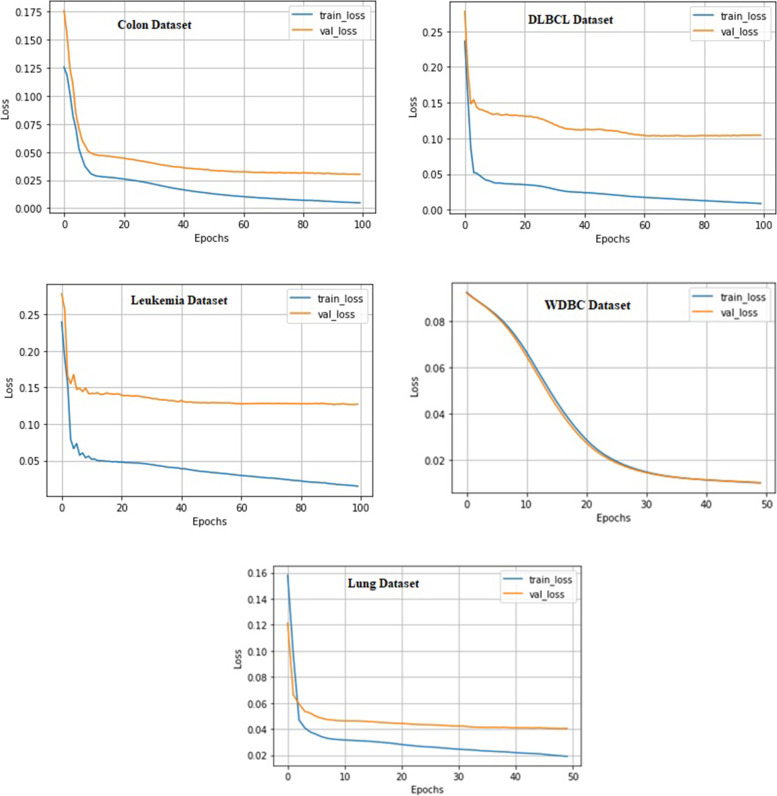
Autoencoder Learning Curves for all used datasets

We build an autoencoder for each dataset. Each autoencoder is trained using the training set and evaluated using the reserved test set. All the parameters of each autoencoder were manually optimized by trial and error. With all datasets, the autoencoders use an exponential decay learning rate with an initial learning rate, decay steps and a decay rate value listed in Table 2. Furthermore, three datasets (colon, leukemia and DLBCL) use 100 epochs, while the WDBC and lung cancer datasets use only 50 epochs. The architecture of each autoencoder is saved with its weights and used later to convert the dimensionality of the reserved test set to the same dimensions as the training set. Figure 7 shows the autoencoder learning curves that resulted for all used datasets. Each curve shows the training loss versus the validation loss with increasing the number of epochs for each dataset. The figure shows the well-fitting of the autoencoder on the WDBC dataset, resulting in the minimum error among all datasets. Increasing the dimensionality of the dataset resulted in increasing the gap between the validation and training curves and hence increasing the loss, as shown in the colon, leukemia, Lung and DLBCL dataset curves.

### Results for all datasets

In this section, the performance of classifiers on each dataset will be discussed. The performance metrics listed in Table 4 have been measured for each classier with each dataset in each of the three evaluation cases. The results for each dataset are summarized in a table containing only the results for classifiers that outperformed their performance with the original and extracted data when they were employed with RN-Autoencoder. The best results have been bolded in each table.

#### Colon dataset

This section discusses the RN-Autoencoder results for the colon dataset. All classifiers that resulted in an increase in their performance in terms of any metric with the colon dataset are listed in Table 5. The results showed that only GNB, AdaBoost and the GB classifiers gained performance when combined with RN-Autoencoder.

**Table 5:** Results summary for the colon dataset

Dataset: colon								
**Classifier**	**Metrics**							
	**Model**	**Test Acc**	**Precision**	**Recall**	**F1**	**Kappa**	**MCC**	**GM**
**GNB**	**Original**	0.6429	0.5	0.6	0.5455	0.2553	0.2582	0.6325
	**RN-SMOTE**	0.5714	0.4	0.4	0.4	0.0667	0.0667	0.5164
	**Extracted**	**0.7857**	**0.6667**	**0.8**	**0.7273**	**0.5532**	**0.5594**	**0.7888**
	**RN-Extracted**	**0.7857**	**0.6667**	**0.8**	**0.7273**	**0.5532**	**0.5594**	**0.7888**
**AdaBoost**	**Original**	0.7143	0.6	**0.6**	0.6	0.3778	0.3778	0.6831
	**RN-SMOTE**	0.6429	0.5	**0.6**	0.5455	0.2553	0.2582	0.6325
	**Extracted**	0.7143	0.6667	0.4	0.5	0.3171	0.3373	0.5963
	**RN-Extracted**	**0.7857**	**0.75**	**0.6**	**0.6667**	**0.5116**	**0.5185**	**0.7303**
**Gradient Boost**	**Original**	0.6429	0.5	0.4	0.4444	0.186	0.1886	0.5578
	**RN-SMOTE**	0.6429	0.5	0.6	0.5455	0.2553	0.2582	0.6325
	**Extracted**	0.7143	0.6667	0.4	0.5	0.3171	0.3373	0.5963
	**RN-Extracted**	**1**	**1**	**1**	**1**	**1**	**1**	**1**

The performance of the GNB Classifier has increased by 14.28, 16.67, 20, 18.18, 29.79, 30.12 and 15.63% in terms of test accuracy, precision, recall, F1, Kappa, MCC and GM scores when used with RN-Autoencoder than when used with original data. Also, it has increased by 21.43, 26.67, 40, 32.73, 48.65, 49.27 and 27.24% in terms of test accuracy, precision, recall, F1, Kappa, MCC and GM scores when used with RN-Autoencoder than when used with RN-SMOTE, while it has not increased when compared with extracted data.

The performance of the AdaBoost classifier has gained an increase of 7.14, 15, 6.67, 13.38, 14.077 and 4.72% in terms of test accuracy, precision, F1, Kappa, MCC and GM scores when used with RN-Autoencoder than when used with original data. However, there is no increase in the recall score. Also, it has increased by 14.28, 25, 12.12, 25.63, 26.03 and 9.78% in terms of test accuracy, precision, F1, Kappa, MCC and GM scores when used with RN-Autoencoder than when used with RN-SMOTE. Finally, when used with the extracted data, it has gained an increase of 7.14, 8.33, 20, 16.67, 19.45, 18.12 and 13.4% in terms of test accuracy, precision, recall, F1, Kappa, MCC and GM scores, respectively.

Finally, the GB classifier with RN-Autoencoder has succeeded in classifying colon cancer with 100% percentage in terms of all metrics. The performance of the GB classifier has gained an increase of 35.71, 50, 60, 55.56, 81.4, 81.14 and 44.22% in terms of test accuracy, precision, recall, F1, Kappa, MCC and GM scores when used with RN-Autoencoder than when used with original data. Also, it has increased by 35.71, 50, 40, 45.45, 74.47, 74.18 and 36.75% in terms of test accuracy, precision, recall, F1, Kappa, MCC and GM scores when used with RN-Autoencoder than when used with the RN-SMOTE. Finally, when used with the extracted data, it has gained an increase of 28.57, 33.33, 60, 50, 68.29, 66.27 and 40.37% in terms of test accuracy, precision, recall, F1, Kappa, MCC and GM scores, respectively.

#### Leukemia dataset

This section discusses the results of RN-Autoencoder with the leukemia dataset. All classifiers that improved their performance with the leukemia dataset in terms of any metric are listed in Table 6. The results showed that SVM-RBF, SGD-LR, KNN and LDA classifiers gained performance when combined with the RN-Autoencoder.

**Table 6:** Results summary for the leukemia dataset

Dataset: leukemia								
**Classifier**	**Metrics**							
	**Model**	**Test Acc**	**Precision**	**Recall**	**F1**	**Kappa**	**MCC**	**GM**
**SVM-RBF**	**Original**	0.6765	**1**	0.2143	0.3529	0.2429	0.3718	0.4629
	**RN-SMOTE**	0.6765	**1**	0.2143	0.3529	0.2429	0.3718	0.4629
	**Extracted**	0.7647	**1**	0.4286	0.6	0.4688	0.5533	0.6547
	**RN-Extracted**	**0.9118**	**1**	**0.7857**	**0.88**	**0.8118**	**0.8266**	**0.8864**
**SGD (LR)**	**Original**	0.6765	**1**	0.2143	0.3529	0.2429	0.3718	0.4629
	**RN-SMOTE**	0.7353	**1**	0.3571	0.5263	0.3953	0.4963	0.5976
	**Extracted**	0.7941	**1**	0.5	0.6667	0.5405	0.6086	0.7071
	**RN-Extracted**	**0.8824**	**1**	**0.7143**	**0.8333**	**0.7463**	**0.7715**	**0.8452**
**KNN**	**Original**	0.7059	0.8333	0.3571	0.5	0.3359	0.3965	0.5825
	**RN-SMOTE**	0.7647	0.7143	0.7143	0.7143	0.5143	0.5143	0.7559
	**Extracted**	0.7647	**1**	0.4286	0.6	0.4688	0.5533	0.6547
	**RN-Extracted**	**0.7941**	0.8889	**0.5714**	**0.6957**	**0.5509**	**0.5817**	**0.7368**
**LDA**	**Original**	0.7059	**1**	0.2857	0.4444	0.32	0.4364	0.5345
	**RN-SMOTE**	0.8235	0.75	0.8571	0.8	0.6434	0.648	0.8281
	**Extracted**	0.7941	0.8182	0.6429	0.72	0.5609	0.5711	0.7606
	**RN-Extracted**	**1**	**1**	**1**	**1**	**1**	**1**	**1**

The performance of the SVM-RBF classifier has gained an increase of 23.53, 57.14, 52.71, 56.89, 45.48 and 42.35% in terms of test accuracy, recall, F1, Kappa, MCC and GM scores when used with RN-Autoencoder than when used with both original data and RN-SMOTE. When used with the extracted data, it has gained an increase of 14.71, 35.71, 28, 34.3, 27.33 and 23.17% in terms of test accuracy, recall, F1, Kappa, MCC and GM scores. Also, The SVM-RBFBF classifier has maintained its precision score value at 100% for the different scenarios.

The performance of the SGD-LR classifier has gained an increase of 20.59, 50, 48.04, 50.34, 39.97 and 38.23% in terms of test accuracy, recall, F1, Kappa, MCC and GM scores when used with RN-Autoencoder than when used with original data.

Also, it has increased by 14.71, 35.72, 30.7, 35.1, 27.52 and 24.76% in terms of test accuracy, recall, F1, Kappa, MCC and GM scores when used with RN-Autoencoder than when used with RN-SMOTE. When used with the extracted data, it has gained an increase of 8.83, 21.43, 16.66, 20.58, 16.29 and 13.81% in terms of test accuracy, recall, F1, Kappa, MCC and GM scores. Also, the SGD-LR classifier succeeded in keeping its precision score value at 100% in the different cases.

The performance of the KNN classifier has gained an increase of 8.82, 5.56, 21.43, 19.57, 21.5, 18.52 and 15.43% in terms of test accuracy, precision, recall, F1, Kappa, MCC and GM scores when used with RN-Autoencoder than when used with original data. Also, it has increased by 2.94, 17.46, 3.66 and 6.74% in terms of test accuracy, precision, Kappa, MCC but decreased by 14.29, 1.86 and 1.91% in terms of recall, F1 and GM scores when used with RN-Autoencoder than when used with RN-SMOTE. When comparing the extracted data, it has gained an increase of 2.94, 14.28, 9.57, 8.21, 2.84 and 8.21% in terms of test accuracy, recall, F1, Kappa, MCC and GM scores and this performance has decreased by 11.11% in terms of the precision score.

Finally, the LDA classifier with RN-Autoencoder has succeeded in classifying leukemia cancer with 100% percentage in terms of all metrics. The performance of LDA classifier has gained an increase of 29.41, 71.43, 55.56, 68, 56.36 and 46.55% in terms of test accuracy, recall, F1, Kappa, MCC and GM scores when used with RN-Autoencoder than when used with original data.

Also, it has increased by 17.65, 25, 14.29, 20, 35.66, 35.2 and 17.19% in terms of test accuracy, precision, recall, F1, Kappa, MCC and GM scores when used with RN-Autoencoder than when used with RN-SMOTE. When used with the extracted data, it has gained an increase by 20.59, 18.18, 35.71, 28, 43.91, 42.89 and 23.94% in terms of test accuracy, precision, recall, F1, Kappa, MCC and GM scores.

#### DLBCL dataset

This section discusses the results for the DLBCL dataset. All classifiers that performed better according to any metric with the DLBCL dataset are listed in Table 7. The results showed that RF, CART, SVM-RBF, AdaBoost and XGBoost classifiers gained performance when combined with the RN-Autoencoder.

**Table 7:** Results summary for the DLBCL dataset

Dataset: DLBCL								
**Classifier**	**Metrics**							
	**Model**	**Test Acc**	**Precision**	**Recall**	**F1**	**Kappa**	**MCC**	**GM**
**RF**	**Original**	0.9167	**1**	0.6667	0.8	0.75	0.7746	0.8165
	**RN-SMOTE**	0.9167	**1**	0.6667	0.8	0.75	0.7746	0.8165
	**Extracted**	0.8333	**1**	0.3333	0.5	0.4286	0.5222	0.5774
	**RN-Extracted**	**0.9583**	**1**	**0.8333**	**0.9091**	**0.8824**	**0.8885**	**0.9129**
**CART**	**Original**	0.9167	0.75	**1**	0.8571	0.8	0.8165	0.9428
	**RN-SMOTE**	0.9167	0.75	**1**	0.8571	0.8	0.8165	0.9428
	**Extracted**	0.9583	**1**	0.8333	0.9091	0.8824	0.8885	0.9129
	**RN-Extracted**	**1**	**1**	**1**	**1**	**1**	**1**	**1**
**SVM-RBF**	**Original**	0.75	0	0	0	0	0	0
	**RN-SMOTE**	0.875	**1**	0.5	0.6667	0.6	0.6547	0.7071
	**Extracted**	0.7917	**1**	0.1667	0.2857	0.2308	0.3612	0.4082
	**RN-Extracted**	**0.9583**	**1**	**0.8333**	**0.9091**	**0.8824**	**0.8885**	**0.9129**
**AdaBoost**	**Original**	0.9583	**1**	0.8333	0.9091	0.8824	0.8885	0.9129
	**RN-SMOTE**	0.9583	**1**	0.8333	0.9091	0.8824	0.8885	0.9129
	**Extracted**	0.9583	**1**	0.8333	0.9091	0.8824	0.8885	0.9129
	**RN-Extracted**	**1**	**1**	**1**	**1**	**1**	**1**	**1**
**XGBOOST**	**Original**	0.9167	**1**	0.6667	0.8	0.75	0.7746	0.8165
	**RN-SMOTE**	0.9167	**1**	0.6667	0.8	0.75	0.7746	0.8165
	**Extracted**	0.9167	**1**	0.6667	0.8	0.75	0.7746	0.8165
	**RN-Extracted**	**0.9583**	**1**	**0.8333**	**0.9091**	**0.8824**	**0.8885**	**0.9129**

The performance of the RF classifier has gained an increase of 4.16, 16.66, 10.91, 13.24, 11.39 and 9.64% in terms of test accuracy, recall, F1, Kappa, MCC and GM scores when used with RN-Autoencoder than when used with both original data and RN-SMOTE. Finally, when used with the extracted data, it has gained an increase of 12.5, 50, 40.91, 45.38, 36.63 and 33.55% in terms of test accuracy, recall, F1, Kappa, MCC and GM scores. The RF classifier has preserved its precision score value at 100% for the different scenarios.

The CART classifier with RN-Autoencoder has succeeded in classifying cancer using the DLBCL dataset with 100% in terms of all metrics. Also, its performance has increased by 8.33, 25, 14.29, 20, 18.35 and 5.72% in terms of test accuracy, precision, F1, Kappa, MCC and GM scores when used with RN-Autoencoder than when used with both original data and RN-SMOTE. Finally, when used with the extracted data, it has gained an increase of 4.17, 16.67, 9.09, 11.76, 11.15 and 8.71% in terms of test accuracy, recall, F1, Kappa, MCC and GM scores. The CART classifier has achieved 100% precision in the cases of extracted data and the RN-Autoencoder.

The performance of the SVM-RBF classifier has gained an increase of 20.83, 100, 83.33, 90.91, 88.24, 88.85 and 91.29% in terms of test accuracy, precision, recall, F1, Kappa, MCC and GM scores when used with RN-Autoencoder than when used with original data. Also, it has increased by 8.33,33.33, 24.24, 28.24, 23.38 and 20.58% in terms of test accuracy, recall, F1, Kappa, MCC and GM scores when used with RN-Autoencoder than when used with RN-SMOTE. Finally, when used with the extracted data, it has gained an increase of 16.66, 66.66, 62.34, 65.16, 52.73 and 50.47% in terms of test accuracy, recall, F1, Kappa, MCC and GM scores, while keeping the precision score at 100% in the two cases.

The AdaBoost classifier with RN-Autoencoder has succeeded in classifying cancer using the DLBCL dataset with 100% percentage in terms of all metrics. AdaBoost performance has gained an increase of 4.17, 16.67, 9.09, 11.76, 11.15 and 8.71% in terms of test accuracy, recall, F1, Kappa, MCC and GM scores with no increase in precision scores when used with RN-Autoencoder than when used with original data, RN-SMOTE and extracted data. The AdaBoost classifier has maintained its precision score value at 100% for all the different scenarios.

Finally, the performance of the XGBoost classifier has gained an increase of 4.16, 16.66, 10.91, 13.24, 11.39 and 9.64% in terms of test accuracy, recall, F1, Kappa, MCC and GM scores when used with the RN-Autoencoder than when used with original data, RN-SMOTE and extracted data. The XGBoost classifier succeeded in keeping its precision score value at 100% in the different cases.

#### Lung (Michigan) dataset

This section discusses the results for the Lung dataset. All classifiers that resulted in an increase in their performance in terms of any metric with the Lung dataset are listed in Table 8. The results showed that GNB, GB and XGBoost classifiers gained in performance when combined with the RN-Autoencoder.

**Table 8:** Results summary for the lung dataset

Dataset: Lung (Michigan)								
**Classifier**	**Metrics**							
	**Model**	**Test Acc**	**Precision**	**Recall**	**F1**	**Kappa**	**MCC**	**GM**
**GNB**	**Original**	0.95	0.9474	**1**	0.973	0.6429	0.6882	0.7071
	**RN-SMOTE**	0.95	0.9474	**1**	0.973	0.6429	0.6882	0.7071
	**Extracted**	**1**	**1**	**1**	**1**	**1**	**1**	**1**
	**RN-Extracted**	**1**	**1**	**1**	**1**	**1**	**1**	**1**
**Gradient Boost**	**Original**	0.95	0.9474	**1**	0.973	0.6429	0.6882	0.7071
	**RN-SMOTE**	0.95	0.9474	**1**	0.973	0.6429	0.6882	0.7071
	**Extracted**	**1**	**1**	**1**	**1**	**1**	**1**	**1**
	**RN-Extracted**	**1**	**1**	**1**	**1**	**1**	**1**	**1**
**XGBOOST**	**Original**	0.95	**1**	0.9444	0.9714	0.7727	0.7935	0.9718
	**RN-SMOTE**	0.95	**1**	0.9444	0.9714	0.7727	0.7935	0.9718
	**Extracted**	**1**	**1**	**1**	**1**	**1**	**1**	**1**
	**RN-Extracted**	**1**	**1**	**1**	**1**	**1**	**1**	**1**

For the GNB and GB Classifiers, their performance has increased by 5, 5.26, 2.7, 35.71, 31.18 and 29.29% in terms of test accuracy, precision, F1, Kappa, MCC and GM scores when used with RN-Autoencoder than when used with both original data and RN-SMOTE, while keeping the recall score at 100% in the two cases.

For the XGBoost Classifier, their performance has gained an increase of 5, 5.56, 2.86, 22.73, 20.65 and 2.82% in terms of test accuracy, recall, F1, Kappa, MCC and GM scores when used with RN-Autoencoder than when used with both original data and RN-SMOTE, while keeping the recall score at 100%.

GNB, GB and XGBoost classifiers have succeeded in classifying lung cancer with 100% percentage in terms of all metrics after pre-processing the dataset using both extracted data and RN-Autoencoder.

#### WDBC dataset

This section discusses the results for the WDBC dataset. All classifiers that enhanced their performance with the WDBC dataset in terms of any metric are listed in Table 9. The results showed that GNB, SVM-Linear, XGBoost and LDA classifiers gained an increase in performance when combined with the RN-Autoencoder.

**Table 9:** Results summary for the WDBC dataset

Dataset: WDBC								
**Classifier**	**Metrics**							
	**Model**	**Test Acc**	**Precision**	**Recall**	**F1**	**Kappa**	**MCC**	**GM**
**GNB**	**Original**	**0.9386**	**0.973**	0.8571	0.9114	0.8647	**0.8688**	0.9194
	**RN-SMOTE**	**0.9386**	**0.973**	0.8571	0.9114	0.8647	**0.8688**	0.9194
	**Extracted**	0.9298	0.925	0.881	0.9024	0.8477	0.8483	0.9188
	**RN-Extracted**	**0.9386**	0.9268	**0.9048**	**0.9157**	**0.8674**	0.8676	**0.9312**
**SVM-Linear**	**Original**	0.9825	**1**	0.9524	0.9756	0.9619	0.9626	0.9759
	**RN-SMOTE**	**0.9912**	0.9767	**1**	**0.9882**	**0.9812**	**0.9814**	**0.993**
	**Extracted**	0.9825	**1**	0.9524	0.9756	0.9619	0.9626	0.9759
	**RN-Extracted**	0.9825	0.9545	**1**	0.9767	0.9627	0.9633	0.986
**XGBOOST**	**Original**	0.9649	**1**	0.9048	0.95	0.9231	0.9258	0.9512
	**RN-SMOTE**	**0.9737**	0.9756	0.9524	0.9639	0.9432	0.9433	0.9691
	**Extracted**	0.9649	0.9524	0.9524	0.9524	0.9246	0.9246	0.9623
	**RN-Extracted**	**0.9737**	0.9535	**0.9762**	**0.9647**	**0.9437**	**0.9439**	**0.9742**
**LDA**	**Original**	0.9649	**1**	0.9048	0.95	0.9231	0.9258	0.9512
	**RN-SMOTE**	0.9561	0.9512	0.9286	0.9398	0.9053	0.9054	0.9501
	**Extracted**	0.9737	**1**	0.9286	0.963	0.9426	0.9442	0.9636
	**RN-Extracted**	**1**	**1**	**1**	**1**	**1**	**1**	**1**

The performance of the GNB classifier has gained an increase of 4.77, 0.43, 0.277 and 1.18% in terms of recall, F1, Kappa and GM scores with a decrease of 4.62 and 0.12% in terms of precision and MCC and no change in test accuracy when used with RN-Autoencoder than when used with both original data and RN-SMOTE. Also, when used with the extracted data, it has gained an increase of 0.88, 0.18, 2.38, 1.33, 1.97, 1.93 and 1.24% in terms of test accuracy, precision, recall, F1, Kappa, MCC and GM scores respectively.

The performance of the SVM-Linear classifier has gained an increase of 4.76, 0.11, 0.08, 0.0707 and 1.01% in terms of recall, F1, Kappa, MCC and GM scores and a decrease of 4.55% in terms of the precision score when used with the RN-Autoencoder than when used with both the original and the extracted data. The SVM-Linear classifier has maintained its test accuracy value of 98.25% in different cases. Unfortunately, with the SVM-Linear classifier, RN-SMOTE exceeded RN-Autoencoder and gained an increase of 0.87, 2.22, 1.15, 1.85, 1.81 and 0.7% in terms of test accuracy, precision, F1, Kappa, MCC and GM scores than when used with RN-Autoencoder.

The performance of the XGBoost classifier has gained an increase of 0.88, 7.14, 1.47, 2.06, 1.811 and 2.3% in terms of test accuracy, recall, F1, Kappa, MCC and GM scores with a decrease in precision score of 4.65% when used with RN-Autoencoder than when used with original data. Also, it has gained an increase of 2.38, 0.08, 0.05, 0.06 and 0.51% in terms of recall, F1, Kappa, MCC and GM scores and a decrease of 2.21% in terms of the precision score when used with the RN-Autoencoder than when used with both the original and the extracted data. When used with the extracted data, it has gained an increase of 0.88, 0.11, 2.38, 1.23, 1.91, 1.93 and 1.19% in terms of test accuracy, precision, recall, F1, Kappa, MCC and GM scores.

Finally, the LDA classifier succeeded in classifying cancer using the WDBC dataset by 100% in terms of all metrics. The LDA performance has gained an increase of 3.51, 9.52, 5, 7.69, 7.42 and 4.88% in terms of test accuracy, recall, F1, Kappa, MCC and GM scores when used with RN-Autoencoder than when used with original data. Also, it has gained an increase of 4.39, 4.88, 7.14, 6.02, 9.47, 9.46 and 4.99% in terms of test accuracy, precision, recall, F1, Kappa, MCC and GM scores when used with the RN-Autoencoder than when used with both the original and the extracted data. When used with the extracted data, it has gained an increase of 2.63, 7.14, 3.7, 5.74, 5.58 and 3.64% in terms of test accuracy, recall, F1, Kappa, MCC and GM scores. The LDA classifier kept the precision score value at 100% for the different scenarios except with RN-SMOTE.

Table 10 summarises which classifiers can perform well on each dataset while also indicating the dimensionality and imbalance ratios of each dataset. RN-Autoencoder enhances the performance of different machine learning classifiers for cancer classification based on high dimensional imbalanced gene expressions datasets. In addition, RN-Autoencoder performs well with different classifiers on cancer subclinical datasets like WDBC dataset.

**Table 10:** The improved classifiers using RN-Autoencoders on different datasets

**RN-Autoencoder + Classifier**														
**Classifiers**														
**Dataset**	**#Features**		**Imbalance Ratio**	**CART**	**GB**	**GNB**	**RF**	**KNN**	**XG-Boost**	**SGD-LR**	**SVM-Linear**	**Ada** **Boost**	**SVM-RBF**	**LDA**
**Colon**	**2000**		**55%**	-	✓	✓	-	-	-	-	-	✓	-	-
**Leukemia**	**7129**		**53.19%**	-	-	-	-	✓	-	✓	-	-	✓	✓
**DLBCL**	**7070**		**32.75%**	✓	-	-	✓	-	✓	-	-	✓	✓	-
**Lung (Michigan)**	**7129**		**11.62%**	-	✓	✓	-	-	✓	-	-	-	-	-
**WDBC**	**30**		**59.38%**	-	-	✓	-	-	✓	-	✓	-	-	✓

### Comparison with current state of art

In this section, we compare the performance of RN-Autoencoder with the performance of the work done in the recent studies mentioned in the related work section using the colon, leukemia, DLBCL and WDBC datasets that are mainly used to evaluate RN-Autoencoder. These studies are Pandit et.al [44], Devendra et al. [28], Menaga et al. [30], Uzma et al. [45], Majumder et al. [33], Bustamam et al. [35], Samieinasab et al. [47], Singh et al. [48] and Bacha et al. [50]. Also, with each comparison we use only the metrics and datasets used by the comparative study.

By comparing the performance of RN-Autoencoder with the best results obtained by FBBO + CNN [28], RN-Autoencoder outperformed it by 2% in terms of the test accuracy, recall, precision and F1 scores for both the leukemia and colon datasets.

**Fig. 8:**
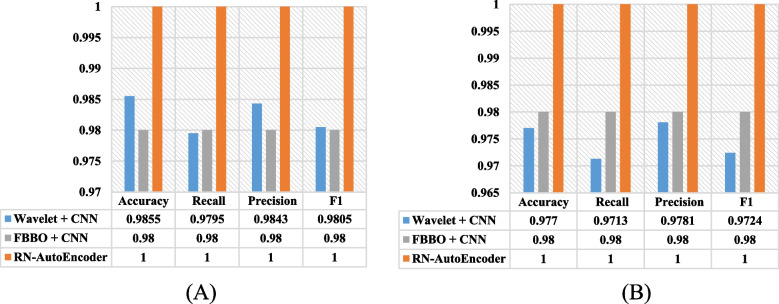
RN-Autoencoder versus FBBO + DCNN and Wavelet + CNN. (A) colon dataset. (B) leukemia Dataset

Also, when compared to Wavelet + CNN [44], RN-Autoencoder outperformed it by 1.45, 2.05, 1.57 and 1.95% in terms of the test accuracy, recall, precision and F1 respectively for the colon dataset. Also, for the leukemia dataset, RN-Autoencoder outperformed by 2.3, 2.87, 2.19 and 2.76% in terms of test accuracy, recall, precision and F1 scores respectively. Figure 8 shows the superior performance of the RN-Autoencoder when compared to both FBBO + CNN and Wavelet + CNN.

**Fig. 9:**
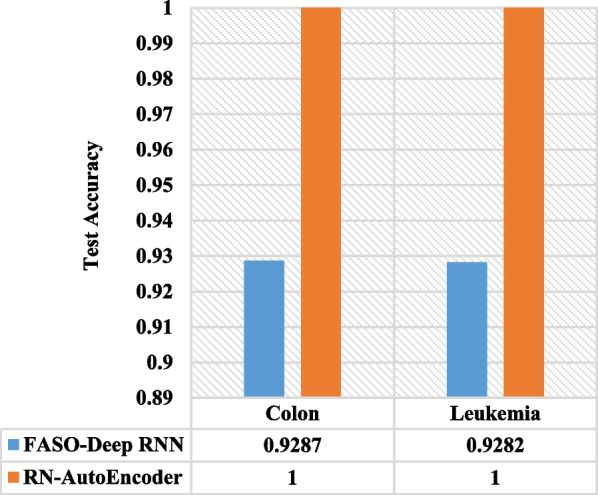
RN-Autoencoder versus FASO-RNN

When comparing RN-Autoencoder to the results of FASO-DEEP-RNN introduced by Menaga et al. [30], RN-Autoencoder outperformed it by 7.13% in terms of the test accuracy with the colon dataset and 7.18% in terms of the test accuracy with the leukemia dataset. Figure 9 shows this comparison.

**Fig. 10:**
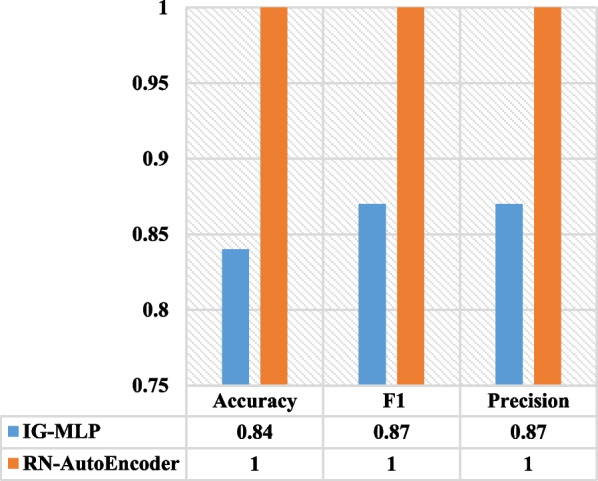
RN-Autoencoder vs IG-MLP

When comparing RN-Autoencoder to the best results obtained by the work done by Majumder et al. [33] using the colon dataset, we find that RN-Autoencoder outperformed it by 16, 13 and 13% in terms of test accuracy, precision and F1 scores respectively as illustrated in Fig. 10.

**Fig. 11:**
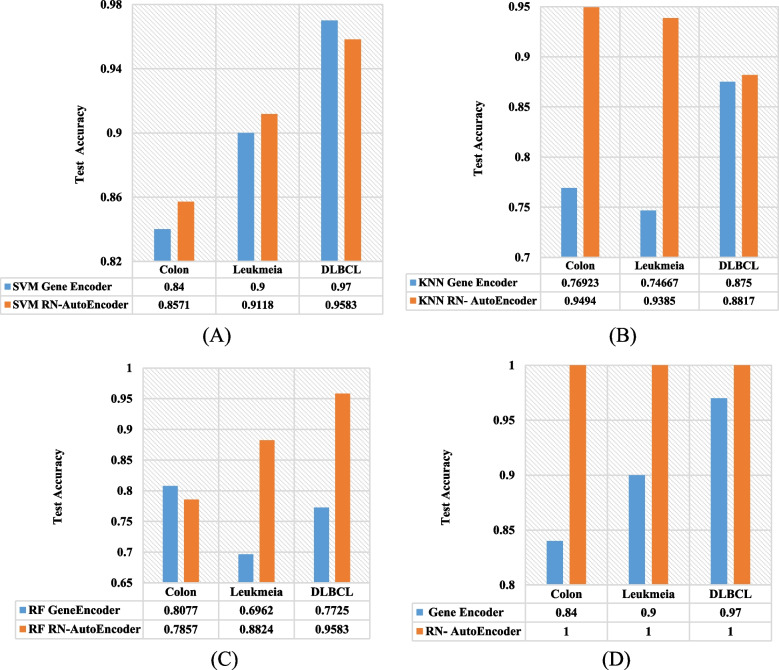
RN-Autoencoder versus Gene Encoder using colon, leukemia and DLBCL datasets: (A**)** SVM Classifier. (B) KNN Classifier. (C) RF Classifier. (D) Best results

Contrasted with Gene-encoder [45], the SVM classifier with RN-Autoencoder outperformed SVM with Gene Encoder by 1.71 and 1.18% in terms of accuracy with colon and leukemia datasets respectively, but lagged by 1.17% for the DLBCL dataset. For the KNN classifier, RN-Autoencoder outperformed Gene-encoder by 18.017, 19.183 and 0.67% for colon, leukemia and DLBCL datasets, respectively. Also, for the RF classifier, RN-Autoencoder outperformed Gene Encoder by 18.62 and 18.58% with leukemia and DLBCL datasets respectively, but lagged by 2.2% with the colon dataset. Since some other classifiers with RN-Autoencoder scored 100% in terms of test accuracy, when this result was compared with the Gene-encoder, RN-Autoencoder outperformed it by 16, 10 and 3% with the colon, leukemia and DLBCL datasets respectively. Figure 11 draws this performance comparison.

**Fig. 12:**
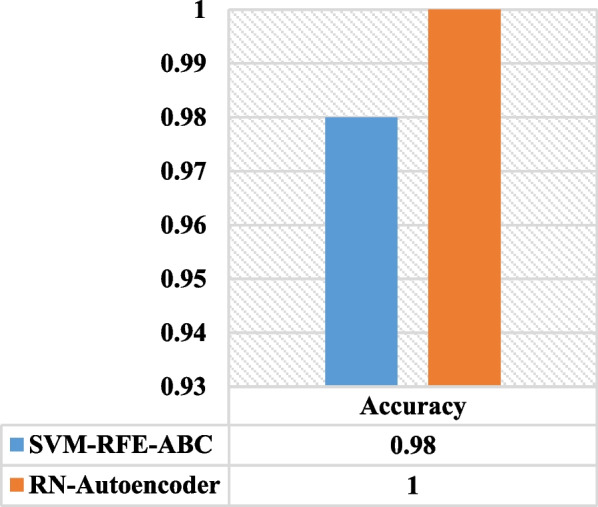
Accuracy of RN-Autoencoder vs SVM-RFE-ABC using Lung (Michigan) dataset

When comparing RN-Autoencoder to the work done in [35] using the lung (Michigan) dataset, we found that the RN-Autoencoder outperformed it by 2% in terms of the accuracy. Also, Fig. 12 shows this comparison.

**Fig. 13:**
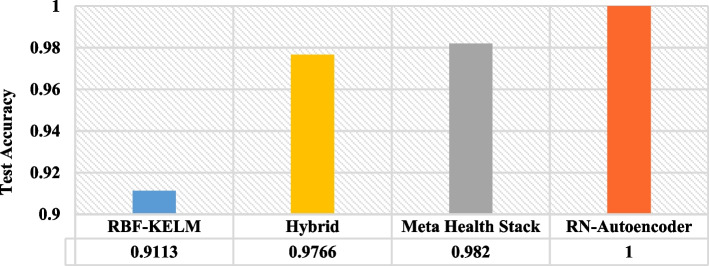
RN-Autoencoder vs other models using WDBC dataset

When comparing RN-Autoencoder to the Meta Health Stack introduced by Samieinasab et al. [47] using the WDBC dataset, we found that the RN-Autoencoder outperformed it by 1.8%, 1.5%, 3.2% and 2.4% in terms of test accuracy, precision, recall and F1 scores respectively. Also, when comparing RN-Autoencoder to the DE-RBF-KELM introduced by Bacha et al. [50] using the WDBC dataset, we found that the RN-Autoencoder outperformed it by 8.87% in terms of test accuracy. Finally, when comparing RN-Autoencoder to the best result obtained by the hybrid work done by Singh et al. [48], we found that the RN-Autoencoder outperformed by 2.34% in terms of test accuracy. Figure 13 shows the comparison between RN-Autoencoder and the mentioned models using the WDBC dataset.

The proposed RN-Autoencoder achieves accurate and precise cancer diagnosis using many imbalanced gene expression datasets. Its performance outperformed the performance of many different recent works. This enhancement mainly depends on the non-linear transformation of the gene expressions using the autoencoder. This is besides the oversampling and noise handling using RN-SMOTE. This combination of steps handles the curse of dimensionality, class imbalance and noise problems that existed in the used datasets and had a bad impact on the performance of many classifiers in recent studies. So, RN-Autoencoder with this sequence of steps improves the performance of many different classifiers in terms of various classification metrics compared to earlier proposals.

## Conclusion and future work

This paper introduced RN-Autoencoder for classifying imbalanced high-dimensional cancer gene expression datasets. RN-Autoencoder utilizes the Autoencoder to reduce the high-dimensionality of the gene expressions and then handle the class imbalance using RN-SMOTE. RN-Autoencoder has been evaluated using many different classifiers and many different imbalanced datasets with different imbalance ratios. The results proved that the performance of the classifiers has been improved with RN-Autoencoder and outperformed the performance with original data and extracted data with percentages based on the classifier, dataset and evaluation metric. Some classifiers succeeded in classifying cancer with 100% performance in terms of all used metrics. In addition, RN-Autoencoder outperformed many recent works using the same datasets. As a future work, RN-Autoencoder will be extended to include more genomic datasets including TCGA with higher dimensionality and multiclass gene expressions datasets. Also, time analysis will be required and then suitable hyperparameter optimization techniques will be applied for RN-Autoencoder to reduce the time for dimensionality reduction and classification.

## Data Availability

The datasets analysed during the current study are available in the Kent Ridge and UCI repositories at the following links: https://web.archive.org/web/20080207153800/http://research.i2r.a-star.edu.sg/rp/. https://archive.ics.uci.edu/ml/datasets/Breast+Cancer+Wisconsin+(Diagnostic).

## Abbreviations

RN-Autoencoder: Reduced Noise Autoencoder
RN- SMOTE: Reduced Noise-Synthesis Minority Over Sampling Technique
DLBCL: Diffuse Large B-Cell Lymphoma
WDBC: Wisconsin Diagnostic Breast Cancer
GBM: Glioblastoma Multiforme
SEER: Surveillance, Epidemiology and End Results
PSO: Particle Swarm Optimization
GA: Genetic Algorithms
PCA: Principal Component Analysis
LDA: Linear Discriminant Analysis
FA: Factor Analysis
ICA: Independent Component Analysis
SVD: Singular Value Decomposition
MDS: Multidimensional Scaling Analysis
KPCA: Kernel-based Principal Component Analysis
NMF: Nonnegative Matrix Factorization
WT: Wavelet Transformation
LLE: Locally Linear Embedding
DBSCAN: Density-Based Spatial Clustering of Applications with Noise algorithm
LR-SMOTE: Limiting Radius SMOTE
SVM: Support Vector Machines
PPCA: Probabilistic Principal Component Analysis
FBBO: Fractional Biogeography-Based Optimization
CNN: Convolutional Neural Network
Deep RNN: Deep Recurrent Neural Network
SGD: Stochastic Gradient Descent
SNP: Single Nucleotide Polymorphism
GEO: Gene Expression Omnibus
ANOVA: Analysis of Variance
IG: Information Gain
MLP: Multilayer Perceptron
KNN: Kth Nearest Neighbours
RF: Random Forest
MIAS: Image Analysis Society
RBF-KELM: Radial-Based Function Kernel Extreme Learning Machines
TP: True positive
FP: False Positive
TN: True Negative
FN: False Negative
MCC: Matthew’s Correlation Coefficient Cohesion’s Kappa
GM: Geometric-Mean
CART: Classification and Regression Tree
GB: Gradient Boost
AdaBoost: Adaptive Boosting
XGBOOST: Extreme Gradient Boosting
GNB: Gaussian Naïve Bayes
SGD-LR: Logistic Regression with Stochastic Gradient Descent
SVM-RBF: Support Vector Machines with Radial Basis Function kernel
SVM-Linear: SVM with Linear kernel
